# Revealing the hierarchical structure of microbial communities

**DOI:** 10.1038/s41598-024-61836-3

**Published:** 2024-05-16

**Authors:** Beatrice Ruth, Stephan Peter, Bashar Ibrahim, Peter Dittrich

**Affiliations:** 1 Department of Mathematics and Computer Science, Friedrich Schiller University Jena, Fürstengraben, 07743 Jena, Germany; 2 Department of Basic Sciences, Ernst-Abbe University of Applied Sciences Jena, Carl-Zeiss-Promenade 2, 07745 Jena, Germany; 3 Department of Mathematics & Natural Sciences and Centre for Applied Mathematics & Bioinformatics, Gulf University for Science and Technology, 32093 Hawally, Kuwait; 4 European Virus Bioinformatics Center, Leutragraben 1, 07743 Jena, Germany

**Keywords:** Microbial communities, Chemical organization theory, Formal concept analysis, Replicator dynamics, High-dimensional data, Environmental microbiology, Computational science

## Abstract

Measuring the dynamics of microbial communities results in high-dimensional measurements of taxa abundances over time and space, which is difficult to analyze due to complex changes in taxonomic compositions. This paper presents a new method to investigate and visualize the intrinsic hierarchical community structure implied by the measurements. The basic idea is to identify significant intersection sets, which can be seen as sub-communities making up the measured communities. Using the subset relationship, the intersection sets together with the measurements form a hierarchical structure visualized as a Hasse diagram. Chemical organization theory (COT) is used to relate the hierarchy of the sets of taxa to potential taxa interactions and to their potential dynamical persistence. The approach is demonstrated on a data set of community data obtained from bacterial 16S rRNA gene sequencing for samples collected monthly from four groundwater wells over a nearly 3-year period (n = 114) along a hillslope area. The significance of the hierarchies derived from the data is evaluated by showing that they significantly deviate from a random model. Furthermore, it is demonstrated how the hierarchy is related to temporal and spatial factors; and how the idea of a core microbiome can be extended to a set of interrelated core microbiomes. Together the results suggest that the approach can support developing models of taxa interactions in the future.

## Introduction

Microbial communities highly adapted to their environment exist nearly everywhere on earth^1,2^. Therefore extending our knowledge on the behavior of those communities helps us to also gain new insights into their environment^2–5^. In order to reveal patterns and processes shaping microbial communities time series analyses are needed^5^. Previous analysis methods mainly focused on external environmental conditions^4,6,7^, the core-microbiome^4,5,8,9^ or pairwise microbial interactions^10–12^. In this context, our new method highlights the internal hierarchical structure of the microbial community. Here it is exemplified on a data set of community data obtained from bacterial 16S rRNA gene sequencing for samples collected monthly from four groundwater wells over a nearly 3-year period (n = 114) along the Hainich Critical Zone Exploratory (CZE)^5^.

The Hainich CZE, a hillslope well transect, is designed to study the formation of a near-surface groundwater microbiome^4,5^. Within the Hainich CZE there are a total of 10 different wells from 2 different aquifers. The data analyzed here originates from four wells further down the hill with 2 wells for each aquifer.

Previously, principal coordinates analysis (PCoA) revealed 6 different microbial community clusters^4^. In that study, through consideration of alpha and beta diversity patterns as well as taxa rank abundance distribution the microbes within the community were subdivided into core and rare taxa^4^. Then core groundwater bacterial taxa, determining the inner community structure, were linked to certain environmental conditions^4^. A follow-up study reflecting upon the temporal dissimilarities of groundwater microbiomes deepened the analysis of the inner structure of the community^5^. Still the used PCoA methods kind of hide the inner structure by projecting the whole structure of each measurement onto a single point in a 2D plane^4,5^.

To better understand the assembly processes determining the local taxonomic formation resulting in the observed community structure we use chemical organization theory (COT) in conjunction with formal concept analysis (FCA). This not only reveals similarities between different communities but also the previously hidden hierarchical structure of the microbial community. This leads to a so-called Hasse diagram exhibiting the different possibilities in a hierarchy of combinations with indications of which measurements contained each set of taxa. In this context, similarities and differences between the different measurements get highlighted. As reoccurring patterns of certain combinations of taxa emerge, the term “core taxa“ needs to be extended to “core communities“. The emergence of reoccurring patterns which unite at least two measurements allows for identifying sets of taxa that form most likely a self-maintaining community in terms of an organization as defined in COT. The complete diagram of measurements and their intersections provides information for deriving interaction rules among taxa in the future.

## Methods

### Chemical organization theory

The new method for revealing the hierarchical structure of a microbial community suggested in this paper is based on the chemical organization theory (COT)^13^. COT assumes a reaction network defined by a set of molecules and a set of reaction rules. COT derives a hierarchical structure consisting of particular subsets of molecules called organizations. An organization is a set of molecules that is closed (i.e., not producing any other molecule outside the set) and self-maintaining (i.e., able to regenerate every molecule used up by reactions within the set). Notably, there is an important, proven link between organizations and non-spatial^13,14^ and spatial dynamics^15–17^. Namely, any long-term behavior must tend towards an organization; in other words, only molecules forming an organization can be persistent^15^.

While previous applications of COT compute organizations and thus potential persistent subsystems for a given reaction network^18–22,27,28^, here the situation is the opposite. Namely, the measurements of potentially persistent states are given, while the reaction network is unknown. In the context of microbial communities, a molecule is equated with a microbial taxon. The reaction network represents supportive interactions among taxa and inflow and decay processes.

**Figure 1:**
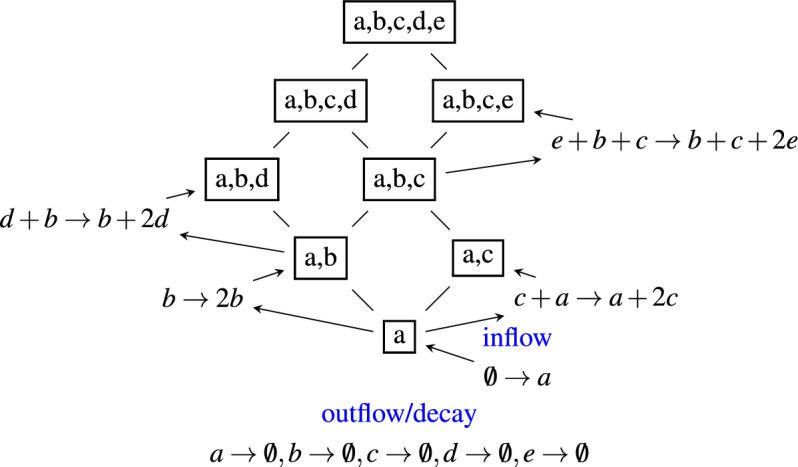
Example of a reaction network (blue) and its Hasse diagram of organizations (black). In this example, there are 5 taxa, $\mathscr {S}= \{a,b,c,d,e\}$. There is an inflow of taxon *a*, represented by reaction “$\rightarrow a$“, having an empty left-hand side. Note that a set of taxa that does not contain *a* is not closed. Thus, taxon *a* must be contained in each organization. Taxon *b* can replicate on its own. Taxon *c* needs *a* to replicate. Taxon *d* needs *b* to replicate. Taxon *e* needs both *b* and *c* to replicate. The full reaction network consists of the following reactions $\mathscr {R} = \{ \rightarrow a, b \rightarrow 2b, c+a\rightarrow a + 2c, a+d \rightarrow a + 2d, e+b+c \rightarrow b +c + 2e, a \rightarrow , b \rightarrow , c \rightarrow , d \rightarrow , e \rightarrow \}$, including a decay reaction for each taxon.

Defining an organization of taxa more precisely, given the set of all taxa $\mathscr {S}$ and a reaction network defined on $\mathscr {S}$ (e.g., Fig. 1), an **organization** $S \subseteq \mathscr {S}$ is a closed and self-maintaining set of taxa^13,23^.

A subset $S\subseteqq \mathscr {S}$ of taxa is **closed** if and only if for each reaction supported by *S* all products of the reaction are contained in *S*. In other words, no active reaction on the subset *S* produces a taxon not contained in *S*. Note that, here, a new taxon can only be created by an inflow (e.g., $\emptyset \rightarrow a$, Fig. 1), and thus every set that contains the inflow is closed.

A subset $S\subseteqq \mathscr {S}$ of taxa is **self-maintaining** if each taxon of *S* is produced by at least one reaction active in *S*, including an inflow reaction. In the example (Fig. 1, the set $\{a, b\}$ is self-maintaing because *a* is produced by the inflow reaction $\emptyset \rightarrow a$ and *b* is produced by self-replication via $b \rightarrow 2b$. The set $\{b \}$ is self-maintaining but not closed. And the set $\{a, d\}$ is closed but not self-maintaining because *d* requires *b* to grow. Note that a simplified definition of self-maintenance can be used due to the particular structure of the reaction network assumed (Assumption 1, below).

All organizations can be arranged in a Hasse diagram (Fig. 1). In a Hasse diagram, the organizations are arranged from bottom to top according to increasing size, that is, the number of taxa they contain. A line is drawn between two organizations if and only if one is a subset of the other and there is no organization between them. This means that the Hasse diagram of organizations gives an overview of all possible combinations of taxa that can be persistent and how they are related.

### Workflow

**Figure 2:**
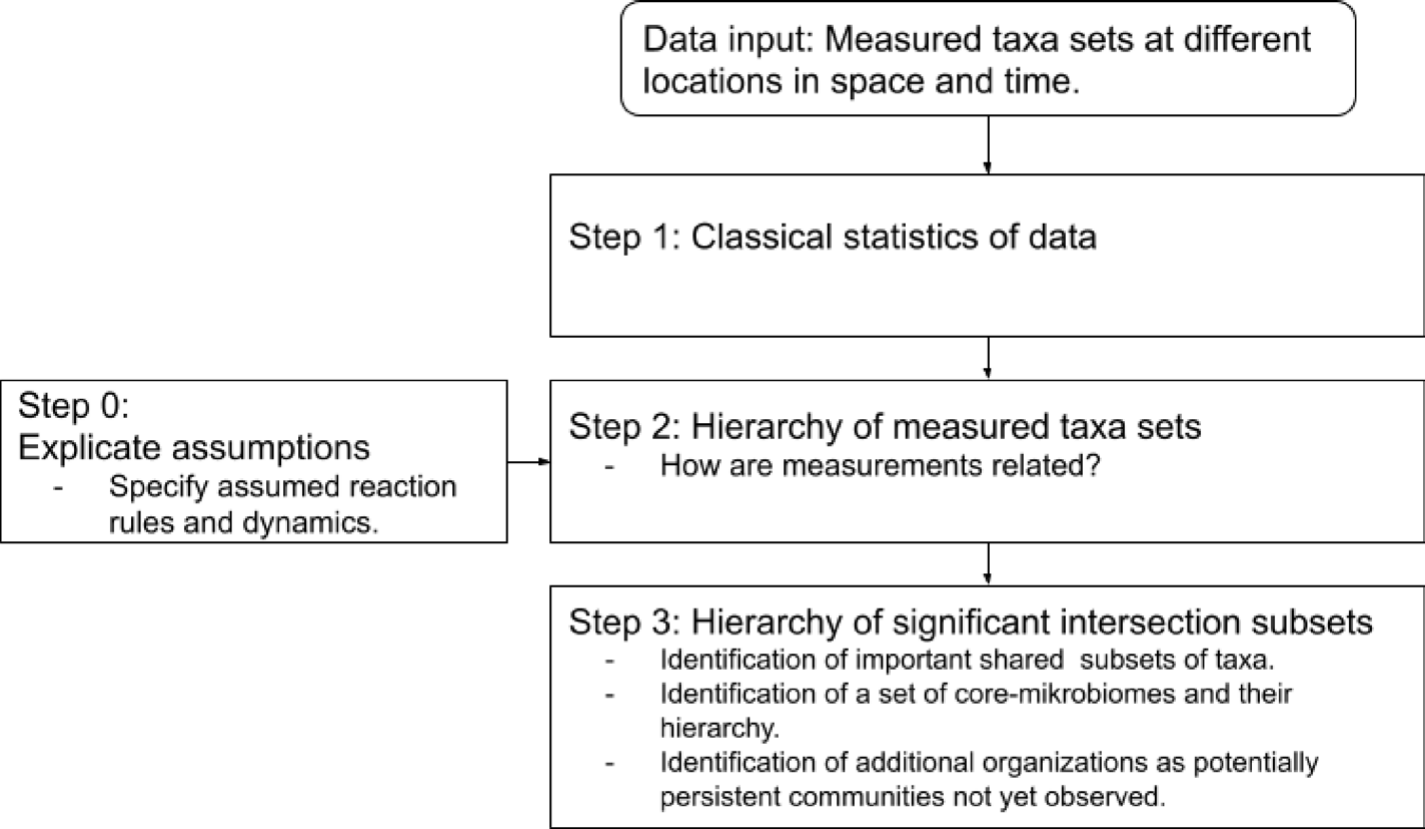
Overview of the method for studying the hierarchical structure of microbial communities.

If the system underlies a generalized replicator dynamics and if each measurement is taken in a stationary or persistent state (Step 0 of the method, Fig. 2) than after statistical testing of the data (Step 1 of the method, Fig. 2) the subset relationships of the measured taxa sets reveal their hierarchical structure (Step 2 of the method, Fig. 2). Through the identification of significant intersection sets (Step 3 of the method, Fig. 2) not only different core-microbiomes but also additional organizations get revealed. Significant intersection sets in this context are for each taxon the group of taxa it was always measured with and intersection sets that are also the union of at least two other measurements.

#### Explicating assumptions (Step 0)

Before performing the actual analyses, the assumptions regarding the dynamics of the microbial communities should be explicated (Step 0 of the method, Fig. 2). Here, the following two assumptions are made:

##### Assumption 1

The (short-term) behavior of the taxa follows a generalized replicator dynamics^24^. This implies that a taxon can only be produced by an inflow (e.g., Fig. 1, reaction rule: $\emptyset \longrightarrow a$. ), by replication (reaction rule: $b \longrightarrow 2 b$), or by a replication necessarily assisted by other taxa (e.g., reaction rule: $e + b + c \longrightarrow b + c + 2e$). Furthermore, replicator dynamics assumes an outflow, that is, all taxa decay spontaneously (e.g., reaction rule: $a \longrightarrow \emptyset$). These assumptions assure (1) that the organizations form an algebraic lattice as a hierarchical structure^13^ and (2) that the set union of two organizations is an organization as well (cf. Fig. 1). Note that the intersection of two organizations is closed but not necessarily self-maintaining.

##### Assumption 2

A measurement is taken in a stationary or persistent state. This implies, together with Assumption 1, that the measured taxa are an organization of the underlying (unknown) interaction network of taxa. This link would later (not in this paper) help derive mechanisms (i.e., interaction or reaction networks) explaining the persistence of communities.

In general microbial interactions can be divided into positive, neutral, or negative^25^. Current COT is only able to reflect upon the positive and neutral interactions. Negative interactions such as competition and amensalism are not directly supported by the theory, yet. This would matter for the hierarchical structure if a negative interaction would cause a species to vanish that would otherwise survive. In this case, the union of two organizations is not necessarily self-maintaining, because a new negative interaction can become effective.

#### Graphical illustration of the hierarchical structure (Step 2 and Step 3)

**Figure 3:**
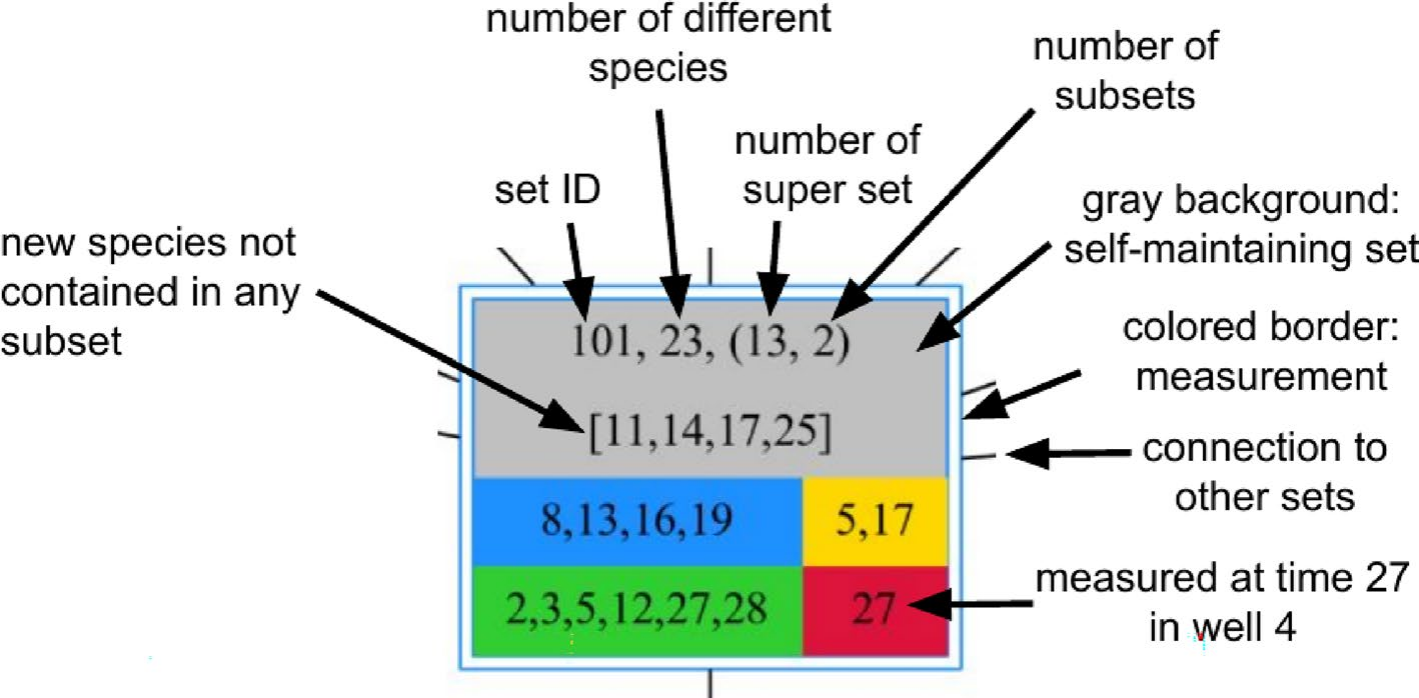
A node in a lattice consists of 2 lines of text followed by a field of four colored boxes representing the different wells with their respective time points. The first line is set ID, the number of different taxa followed in brackets by the number of present supersets and the number of present subsets. The second line enlists all taxa that were not present in any of its considered subsets. The colored border marks the set as a measurement, while the grey background highlights the set as self-maintaining.

Opposing to lattices formed by given reaction systems $<\mathscr {M}, \mathscr {R}>$ it remains unclear if the resulting lattice from the data is complete. The formed lattice depicts the subset relationships between all considered taxa sets. In order to highlight the differences between the individual taxa sets of the lattice nodes were provided with additional information (see Fig.  3).

#### Hierarchy of significant intersection subsets (Step 3)

As the here presented method relies on the formation of all intersection sets the effects of intersection and union on chemical organizations need to be investigated.

##### Theorem 1

The intersection of two organizations leads to a closed set if all molecules can only be produced if they already exist.

##### Proof

This means the condition of self-maintenance gets lost while the condition of seclusion remains.

a Loss of self-maintenance. If there are multiple ways to produce a certain molecule the intersection of two organizations is not necessarily self-maintaining. Example: Reaction system $(\mathscr {M}=\{A,B,C\}, \mathscr {R}=\{A \rightarrow 2A, B \rightarrow 2B, A + C \rightarrow 2C, B + C \rightarrow 2C, C \rightarrow \emptyset \}$ with organizations $\{\{\emptyset \}, \{A\}, \{B\}, \{A,B\}, \{A,C\}, \{B,C\}, \{A,B,C\}\}$ shows that the intersection of the organization $\{A,C\}$ and the organization $\{B,C\}$ , being molecule type C, is not self-maintaining.
b Preservation of seclusion. As the reaction system $(\mathscr {M},\mathscr {R})$ only produces molecules that are already there, the intersection of organizations $\mathscr {O}1$ and $\mathscr {O}2$ will not allow the formation of any new molecule. Molecules $x \in \mathscr {M}$ that are only part of organization $\mathscr {O}1$ and vice versa organization $\mathscr {O}2$ have no reaction $r \in \mathscr {R}$ to produce *x* in each other organization. So even the full set of molecules of the other organization is not enough to produce *x*. So those molecules *x* also will not be produced by the intersection of $\mathscr {O}1$ and $\mathscr {O}2$. Therefore the intersection of the two organizations is closed.

$$\square$$

##### Theorem 2

The union of two organizations leads to a self-maintaining set if no inhibitions are part of the reaction system $(\mathscr {M},\mathscr {R})$.

##### Proof

This means the condition of seclusion gets lost while the condition of self-maintenance remains.

a Loss of seclusion. If a new molecule type $x \in \mathscr {M}$ is added to an organization $\mathscr {O}$ even more molecule types $y \subset \mathscr {M}$ can be produced. Example: Reaction system $(\mathscr {M}=\{A,B,C\}, \mathscr {R}=\{A \rightarrow 2A, B \rightarrow 2B, A + B \rightarrow 2C, C \rightarrow \emptyset \})$ with organizations $\{\{\emptyset \}, \{A\}, \{B\}, \{A,B,C\}\}$ shows that the union of the organization $\{A\}$ and $\{B\}$ is not closed as the new possible reaction $A +B \rightarrow 2C$ also produces the molecule type C.
b Preservation of self-maintenance. As the reaction system $(\mathscr {M},\mathscr {R})$ is without inhibitions the union of organizations $\mathscr {O}1$ and $\mathscr {O}2$ allows no additional reaction reducing the production of any of the present molecules. All molecule types $x \subset \mathscr {M}$ that is needed for the self-maintenance of another molecule type $y \in \mathscr {M}$ of the same organization $\mathscr {O}1$ will also be in the union with another organization $\mathscr {O}2$.

$$\square$$

**Formal Concept Analysis (FCA)** Formal concept analysis studies the mathematical derivation of formal concepts from binary data, called a formal context^26^. In our case, a concept is a pair of a certain set *S* of taxa and a set of measurements that all share the same set *S* of taxa. These concepts can be computed by intersecting the measurements as it is done in Sect.“Significant Subsets”.

#### Code availability

All code used to reproduce the presented subset relationships of the measurements and their intersections is publicly available at https://git.uni-jena.de/ne78xoy/focusedfca.

### Data set from Hainich CZE

The Hainich CZE well transect monitors a hillslope groundwater flow system with two aquifers^4,5^. Groundwater samples were collected monthly from four groundwater wells over a nearly 3-year period resulting in $n=114$ samples. The bacterial taxa within the samples were sequenced through 16S rRNA gene sequencing and classified with the mothur-formatted SILVA v. 132 taxonomy reference database, using the default bootstrapping algorithm (cutoff value: $80\%$, see Table 1)^4^.

**Table 1:** Identified bacterial phyla ordered according to their abundance.

Abundance rank/ Taxon	Phyla	Abundance rank/ Taxon	Phyla	Abundance rank/ Taxon	Phyla
0	Proteobacteria	17	Methylomirabilota	34	WPS-2
1	Patescibacteria	18	Latescibacterota	35	NKB15
2	Nitrospirota	19	Spirochaetota	36	RCP2-54
3	Planctomycetota	20	Margulisbacteria	37	SAR324_clade
4	Bacteria	21	Cyanobacteria		(Marine_group_B)
5	Chloroflexi	22	Firmicutes	38	Fusobacteriota
6	Actinobacteriota	23	WOR-1	39	FCPU426
7	Bacteroidota	24	Dependentiae	40	Calditrichota
8	Verrucomicrobiota	25	Zixibacteria	41	Thermotogota
9	Myxococcota	26	Campilobacterota	42	Schekmanbacteria
10	Desulfobacterota	27	Fibrobacterota	43	Sumerlaeota
11	Acidobacteriota	28	GAL15	44	Firestonebacteria
12	Bdellovibrionota	29	Nitrospinota	45	Entotheonellaeota
13	DTB120	30	Sva0485	46	Deferrisomatota
14	Elusimicrobiota	31	NB1-j	47	TA06
15	Gemmatimonadota	32	Hydrogenedentes	48	WS4
16	MBNT15	33	Armatimonadota	49	PAUC34f

**Figure 4:**
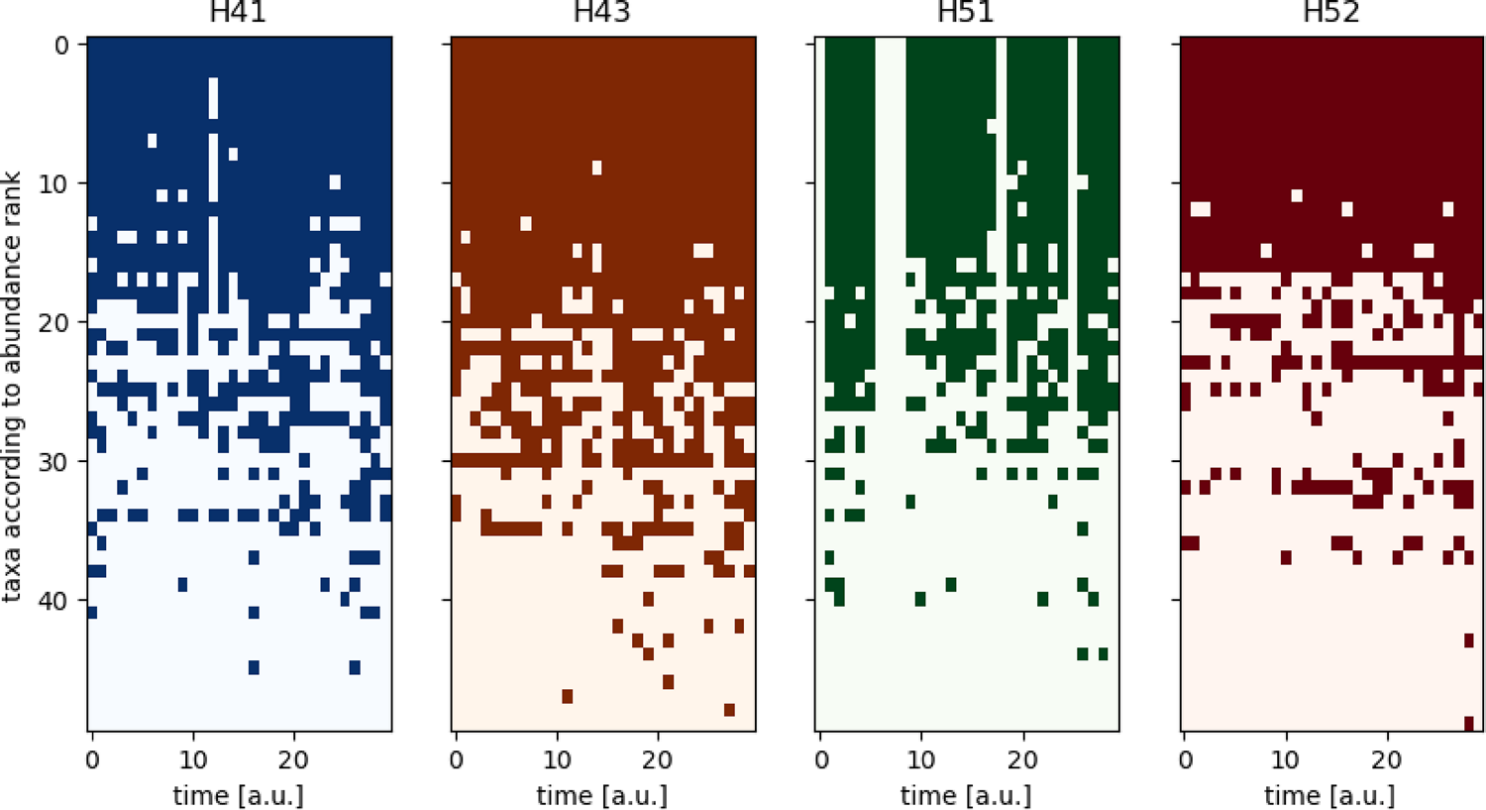
Visualization of the complete data set used here, taken from Ref. ^5^. The data consists of measurements (columns) of the four different wells at 30 different points in time over a period of approximately 30 months. A dark dot indicates that the respective taxon (vertical axis) is present at that point in time (horizontal axis). The blank vertical lines in the illustration for well H51 represent missing data. The time unit is approximately one month.

The given 50-dimensional vectors of phyla from four different wells H41, H43, H51, and H52 measured at 30 different time points (except for well H51 with 6-time points missing) were analyzed. Figure 4 illustrates the binarized measured data. Each taxon is refered to according to its number in Table 1.

## Results

The result section follows a typical sequence of a data analysis process. It starts with a general analysis of the data using classical statistical testing (Sect. “Classical statistical characteristics of the data” ). Then the hierarchical structure formed by subset and superset relationships of the measurements is presented (Sect. “Hierarchy of Measurements” ). Subsequently, this hierarchical structure is complemented by the intersections of the measurements (Sect. “Significant Subsets” ). Because there are many such intersections (15,390), those intersections are identified that introduce new taxa (Sect. “Significant Subsets” ), which can be seen as elementary building blocks of the hierarchy. Furthermore, specific, more complex intersections (called additional organizations) are identified that are also unions of measurements (Sect. “Significant Subsets” ). Finally, the number of required measurements to obtain information about the hierarchical structure is estimated by simulated virtual experiments (Sect. “Virtual Simulated Experiments” ).

### Classical statistical characteristics of the data

**Figure 5:**
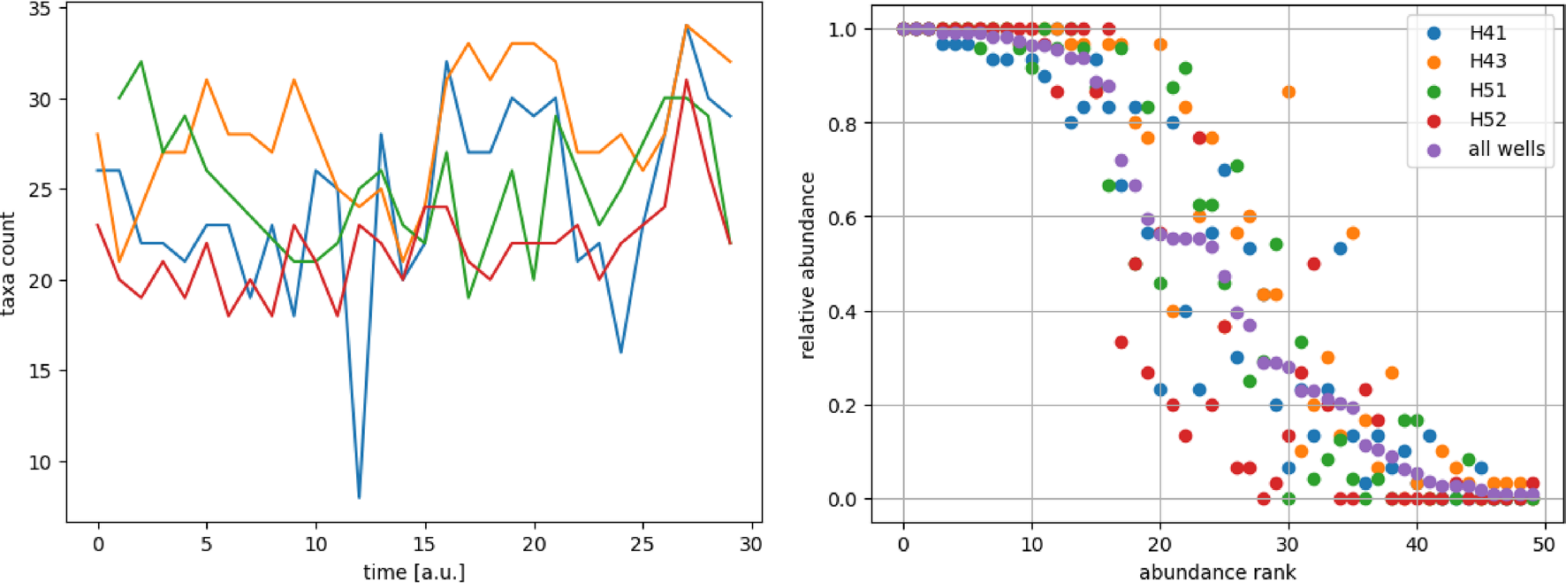
Left: Number of taxa of the four different wells at 30 different points in time. Right: Relative abundance of the individual taxa, represented by their abundance rank, according to the considered well. Each well shows the preferences of certain taxa compared to all measurements (purple).

Starting with a general view of the binarized data (Fig. 4), the four individual tables show that if a taxon is often present in one well, it will likely be highly abundant in the other wells, too (taxa 0-15). On the other hand, some taxa are only present in one well, for example, taxa 47, 48, and 49. It is also observed that certain taxa tend to prefer different wells (Fig. 5).

Over the course of time, there are fluctuations in the taxa counts (see Fig. 5). A measurement consists of 8 (well H41, time point 12) up to 34 (wells H41 and H43, time point 27) different taxa. For well H43, those fluctuations seem to impose a temporal pattern of increasing (time intervals 4-9 and 16-20) and shrinking (time intervals 11-15 and 21-25) taxa diversity. For all performed tests a significance level $\alpha = 0.05$ is assumed. The general difference in the number of taxa between the different wells is statistically evident (Fig. 5 and Table 2). Well H43 has on average a significantly larger number of different taxa present in a measurement compared to the average of all wells whereas well H52 has on average a significantly lower number of different taxa present in a measurement (Table 2: one sample t-test for the number of different taxa).

**Table 2:** Statistical testing.

	all together	H41	H43	H51	H52
average number of different taxa	24.9	24.3	28.2	25.4	21.8
standard deviation	4.5	5.2	3.5	3.6	2.6
$H_0$	Each well has the same distribution in the number of different taxa.				
One sample T-test	–	0.555	$2.17e-05$	0.675	$3.68e-07$
$H_0$	Each well has the same unimodal frequency distribution.				
Two sample Kolmogorov-Smirnov Test	–	0.717	0.396	0.179	0.068
Hartigans’ Dip Test	$8.15e-06$	–	–	–	–
$H_0$	abundance rank ordering is the same in every well.				
Spearman rank-order correlation coefficient	–	0.971	0.972	0.968	0.947
$H_0$	Taxa have the same abundance across different wells				
Chi-square Test	–	0.021	$2.06e-05$	0.039	$3.67e-07$
highest abundance deviation below average	–	20	21	30	22
One sample T-test	–	0.0002	0.104	0.0	$2.78e-07$
highest abundance deviation above average	–	34	30	22	32
One sample T-test	–	0.001	$3.47e-10$	$1.9e-06$	0.007

The frequency distribution of taxa occurrences is the same in every well, as the minimal p-value is $p = 0.068$, and differs significantly from a unimodal distribution, $p=8.15e-06$ (see Table 2: Two sample Kolmogorov-Smirnov Test and Hartigans Dip Test). Together with the histogram (not visualized here) of the frequency distributions one can conclude that for all environments taxa can be either highly abundant or rare. The strong positive correlation between the individual orderings compared to the overall ordering reveals that in general a taxon that is highly present in one well has also a high abundance in the other wells as well. So based on the pure ordering of the abundance rank of the taxa there is no significant difference between the wells as the different orderings are highly connected.

The relative abundance of each individual taxa reveals that each well tends to have certain taxa significantly more or less likely present compared to their average abundance, with maximal p-value $p = 0.039$ for well H51 (see Fig. 5 and Table 2). Only well H43 possesses taxa being significantly rarer than the average as the highest abundance deviation achieved for taxa 21 has $p=0.104$. On the other hand, the highest abundance deviations above average in each well are statistically significant. It can be concluded that the abundance of the taxa 20, 22, 30, 32, and 34, measurements of the four wells can be statistically distinguished.

### Hierarchy of measurements

The first step for revealing the hierarchical structure is to study the subset relationship of all measurements. For each pair of measurements, it is determined whether one is a subset of the other with respect to the measured taxa.

The result can be visualized as a Hasse diagram (Supplement Fig. S3.3), containing 114 nodes, each representing a different set of taxa. Here, there is one node for each measurement because each measurement consists of a unique combination of taxa. The diagram reveals many subset relationships between the measurements. Although the same set of taxa has never occurred twice across space and time, there is a high chance that a sub or superset was also measured for one measured taxa set. Exceptions are measurements in well H41 at time points 9, 23, and 26, in well H43 at time points 7 and 8, in well H51 at time points 23 and 26, and in well H52 at time point 17, which are neither a subset nor superset of other measurements.

#### Detailed set connections of a selected measurement

Although the Hasse diagram of all measurements provides a complete picture of all subset relationships of measured taxa sets, its complexity makes it hard to study individual relations. Therefore, the new method allows to select a particular measurement to investigate its relation to other measurements.

**Figure 6:**
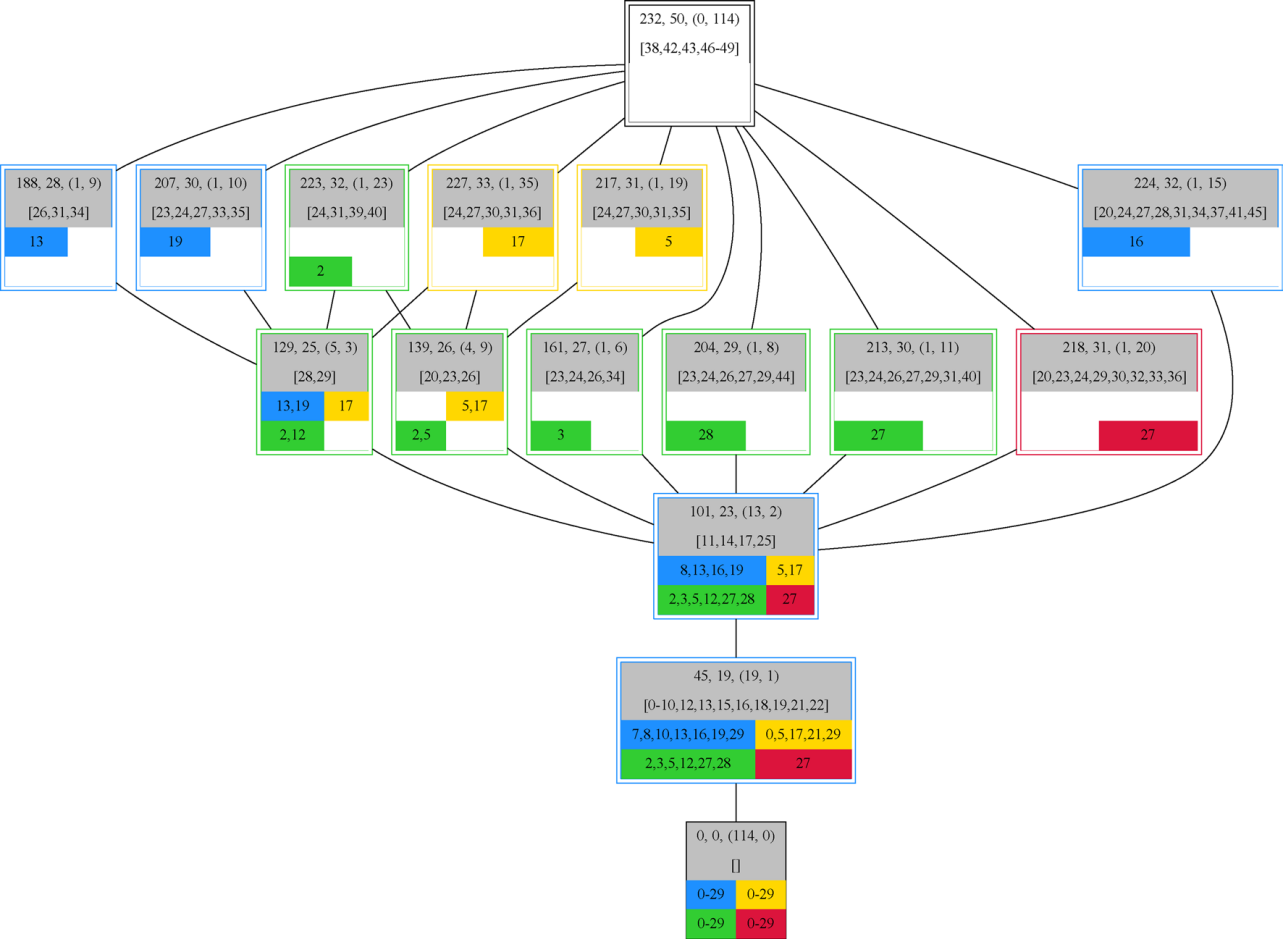
Detailed depiction of sub and superset relationships of measurement 8 in well H41 (set-ID 101). A node representing a distinct set of taxa consists of an identification number, followed by taxa count and the number of sets it is sub and superset of, in parenthesis, and a list of taxa that were not present in any of its subsets. The bottom of each node consists of 4 fields corresponding to the four different wells H41(blue), H43(yellow), H51(green), and H52(red) containing the measurements it is part of. The taxa set associated with set-ID 101 is typical for the blue (H41) and green (H43) well suggesting that the environmental conditions are more beneficial in these wells compared to the other two.

For example, when selecting measurement 8 in well H41 Fig. 6 can be obtained. This measurement consists of a set of 23 taxa and is represented by the central node (taxa-set ID 101). It can be seen that this measurement is a (proper) subset of 12 measurements and a (proper) superset of one measurements.

Taxa that are not present in the real subset measurement (taxa set 45) are 11, 14, 17, and 25 originating also from well H41 (blue border) with possible time points 7, 10, or 29. Getting to the supersets it is observable that the taxa set 101 was found 4 times in well H41 (blue), 2 times in well H43 (yellow), 6 times in well H51 (green), and one time in well H52 (red). Probably the environmental differences are the driving force for the rarity of taxa set 101 in the red well. Also, the yellow well is only represented twice in taxa set 101. But if we look at taxa set 227 and 217, the two measurements of the yellow well, it is observable that they differ only in four taxa. Moving on to the supersets measured in the blue well it is observable that the taxa set 101 was measured 4 times starting at time point 8 and leaving at time point 19 with temporal distances of 5, 3, and 3 measurements. It is also worth noting that taxa set 224 (well H41 time point 16) shares one more taxa with taxa set 188 (well H41 time point 13) and taxa set 207 (well H41 time point 19) than taxa set 188 and 207. Following the temporal order of the measurements in the green well H51 the similarity in the measured taxa sets is lowest for time points 5 and 12 with 23 taxa they have in common and highest for time points 27 and 28 with 28 taxa they have in common. Additionally, the small temporal distance between the first three and last two measurements indicates that the observed taxa sets are stable under the present environmental conditions.

#### Overall connectivity of the measurements

**Figure 7:**
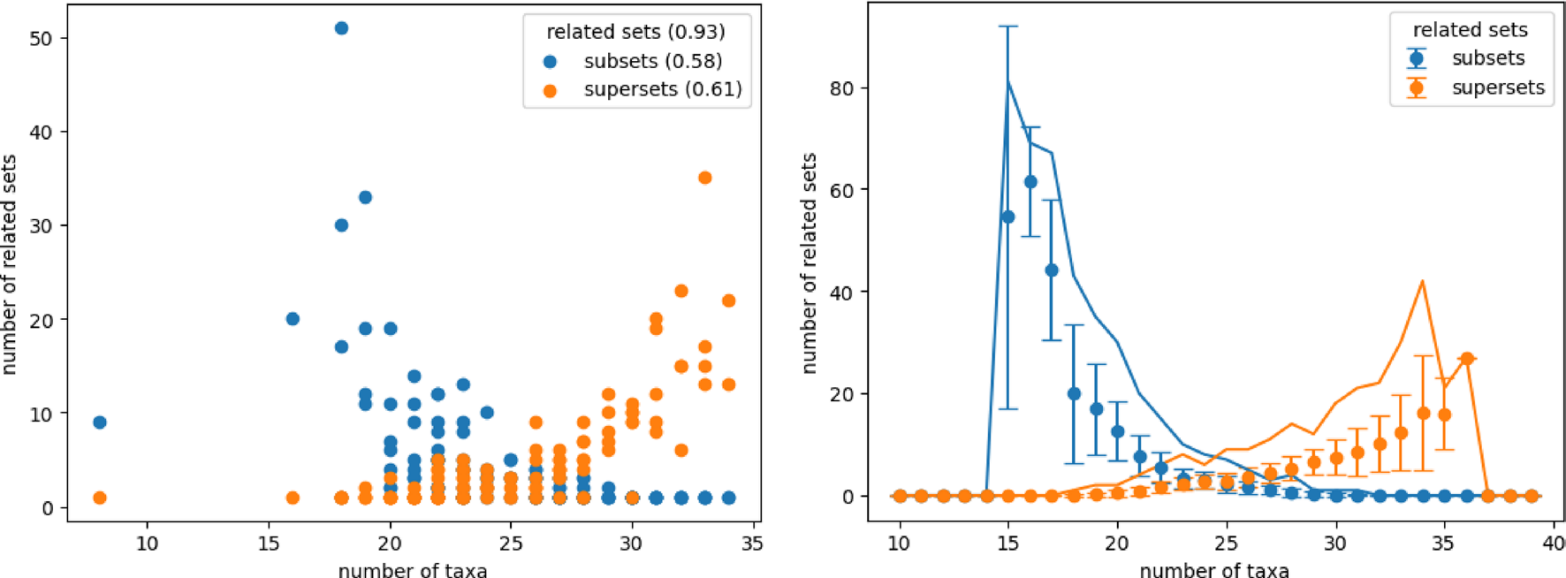
Number of related measurements, actual measurements on the left and artificial measurements on the right, which possess the considered measurement as sub (blue) or superset (orange). Although there is an overall trend that with increasing set size the number of being a subset shrinks while the number of being a superset rises, neither the smallest nor the largest taxa set have the most related sets in the actual measurements (left). Contrary the artificial measurements tend to have the minimal and maximal taxa set as their most connected set (right). Still, actual and artificial measurements share a common feature as the most common subset tends to be larger than the most common superset.

Overall over $90\%$ ($93\%$) of the actual measurements possess at least one other measurement as a subset ($61\%$) or are a subset of at least one other measurement ($58\%$). The number of taxa is thereby no indicator for the number of supersets (see Fig. 7). It is observable, especially for artificial measurements, that it is easier for a small number of taxa to form a subset of many other measurements than for a great number of taxa to represent a superset of many other measurements.

The highest number of being subset is reached for a measurement of 18 taxa and those 18 taxa are also present in 50 other measurements, which corresponds to nearly half (approx 0.44) of the total amount of the 114 measurements. This combination of 18 taxa is very redundant hence there exist important interactions between those bacteria which support the viability of their surrounding. From that on the minimal measured taxa sets can be seen as a kind of main taxa which have the potential to have additional taxa represented by other measurements.

On the other hand, there is a measurement with 33 different taxa which is a superset of 34 other measurements. This most common superset represents a combination of taxa that can be subdivided into the most other measurements. Thus there are many combinations of taxa subsumed making this taxa set to a kind of general taxa. On one hand, it supports the measured subsets and on the other hand, reveals that those combinations can also coexist in a greater context. Even though the overall absolute maximal number of connections is in the median range of that of the artificial measurements, the number of taxa differs. Actual measurements have more taxa in their most connected subset and fewer taxa in their most connected superset than artificial measurements.

#### Up-building taxa sets represented by measurements

**Figure 8:**
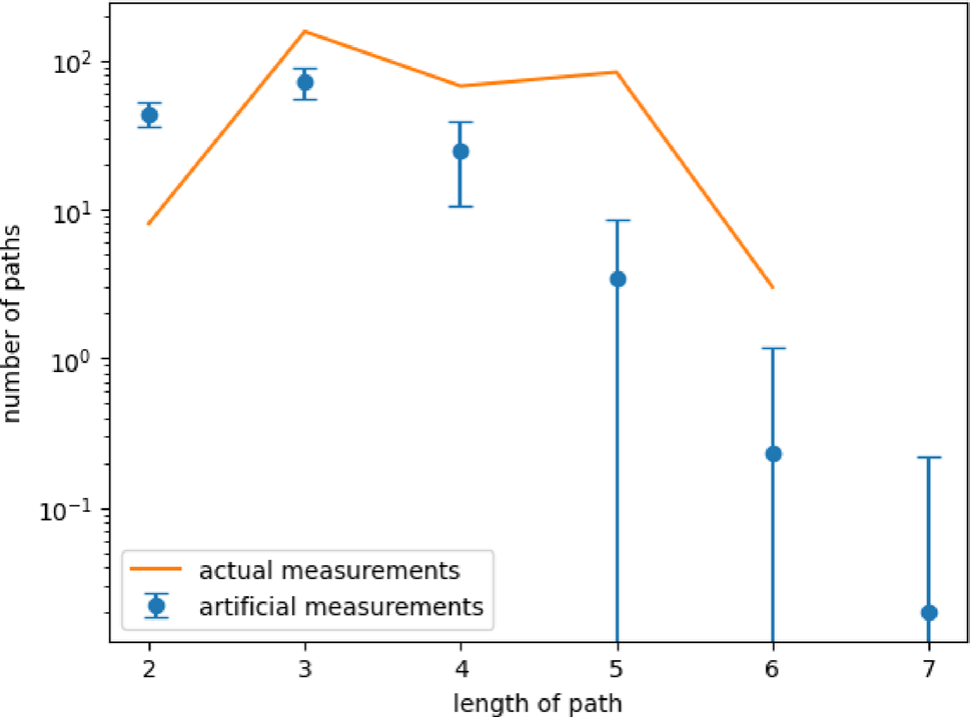
The different levels of the measured subset relations of the corresponding Hasse diagram of the measurements.The length of the path describes how many edges are inbetween the empty set and the set of all taxa. A length of path of 2 describes a scenario in which a single measurement is directly connected with both of those sets, the empty set and the set of all taxa. So it is neither super nor sub-set of any other measurement. On the other hand, a path length of 6 states that there is a follow-up of 5 measurements such that each is a subset of the next.

To examine the number of up-building specie sets of the Hasse diagram of the measurements (see Supplement Fig. S3.3) the 317 different paths from the empty set to the set of all taxa were analyzed (see Fig. 8). The shortest length of 2, with 8 occurrences, indicating only one measurement in between the empty set and the set of all taxa shows the measurements which do not possess any other measurement as a subset or superset. By far most amount of paths (49%) have just one further measurement included whereas the longest path of length 6, with 3 observations, contains a follow-up of 5 measurements. The observed distribution of the lengths of paths indicates that a succession of growing taxa sets is limited. Such a succession is not easily achievable as therefore a certain set of taxa has to be expandable in a row of intermediate steps. So it is interesting to see that still, such a high amount of paths (83) has a follow-up of 4 measurements. Contrary to the set connectivity the path length was not that good replicable by the artificial measurements (see Fig. 8). Although artificial measurement sets have the potential to obtain a maximal path length of eight it is most likely to obtain paths of length two to four which is not the case for the actual data where path lengths of 3 to 5 are most common. Especially the difference in the amount of path length two tells actual and artificial measurements apart. It seems that up-building taxa sets are not easily replicated by artificial measurements leading to the assumption that there is an inherent characteristic to the data making a path length of two less likely in terms of allowing for a follow-up of different addable taxa.

### Significant subsets

Formal concept analysis (FCA) as a conceptual clustering technique would suggest deriving all intersections of all measurements to identify the different concepts of the data. This would lead to a total of 15390 different taxa sets. As mentioned in the method section (see methods Sect. “Methods”) there are two groups of intersections with special interest. On the one hand, those taxa sets introducing new taxa that are not present in any of their subsets, 42 taxa sets, and on the other hand those taxa sets that are also the union of at least two other measurements, representing 75 additional organizations. In 100 artificial measurement sets of 120 measurements, $70148.89 \pm 31426.91$ different intersection sets including $44.48 \pm 1.79$ taxa sets introducing new taxa and $13.32 \pm 8.11$ additional organizations were computed. Contrary to the total amount of intersection sets and taxa sets introducing new taxa additional organizations in the actual measurements are very high compared to the average of 100 artificial measurements.

#### Subsets introducing new taxa

We want to focus first on the subsets introducing new taxa which are of great interest as they reveal those taxa combinations that are most common and reveal the smallest combination of different taxa a certain taxon had along its own appearance (see Supplement Fig. S4.4). An alternative depiction is achieved through an upset plot (see Supplement Fig. S4.5). Instead of the concrete taxa combinations, the focus is on the similarity between the different measurements, such that it remains unclear which three taxa are represented by all measurements. In contrast, our graph reveals taxa 0-3(see Supplement Fig. S4.4). On the other hand, the intersection set with the most taxa contains 25 taxa. Counterintuitively artificial measurements possess even more taxa in their biggest intersection sets with up to 33 taxa still occurring 6 times.

**Detailed set connections on an individual set of taxa**

**Figure 9:**
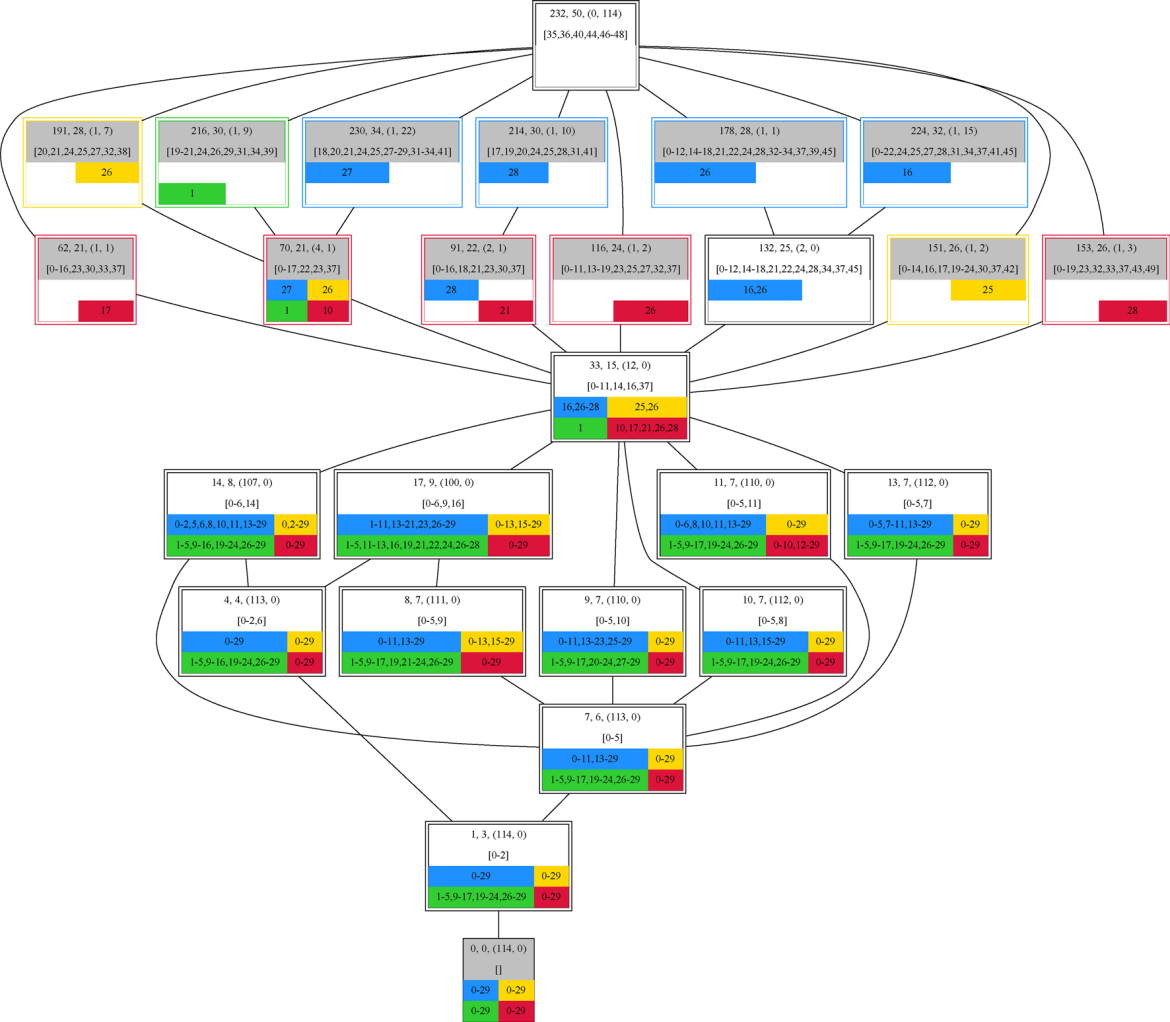
Set relationships of taxa set 33 (central node) which is a subset of 12 measurements (on the node’s first line, first number in round brackets) introducing taxon 37 (node’s second line, last number in square brackets). This subset of taxa is mostly present in the red well (H52) (red box with time points 10, 17, 21, 26 and 28) and measured only once in the green well (H51) (green box with time point 1). This set of taxa is only closed (white background) as neither the set itself nor a combination of its subsets is a complete measurement. But it has to be pointed out that the blue, yellow, and red well have all this taxa combination in their measurements at time point 26 in common.

As an example, we want to look at taxa set 33 which is a subset of 12 measurements. With regard to the outlay of the lattice we also obtain the measurements of the individual wells which possess this combination. This set is introducing taxon 37 which only occurred once in well H51 (green) and twice in well H43 (yellow) but four and 5 times in well H41 (blue) and well H52 (red) respectively. The common part shared between those 12 measurements are taxa 0-11, 14, 16, and 37. It is observable that the connected smaller intersection sets are shared between much more measurements. Set 17 is thereby the minimum with only 100 measurements possessing taxa 0-6, 9, and 16. This corresponds to $87.7\%$ of the measurements.

**Core microbiome** Previous computation methods would declare every taxon occurring in more than $90\%$ of the measurements, here 103 measurements, as part of the core-microbiome. So taxa 0 to 14 would form the core-microbiome. In the lattice now several sets of different taxa combinations make up 11 different core-microbiomes each full filling the condition of being measured at least 103 times (see Supplement Fig. S4.4). The biggest core-microbiome using this method includes 8 taxa (0 to 6 and 14) which are part of 107 measurements. Taxa 7 to 14 are thereby represented only once each in a different core-microbiome. The set directly above the empty set corresponds to the smallest core-microbiome, here taxa 0-2, as those taxa are present in every measurement. Comparing these findings with core-microbiomes obtained in artificial measurements there were in total 390 different cores with each measurement set having $12.83 \pm 1.183$ different core-microbiomes. Here the biggest core-microbiome consists of 9 different taxa, occurring in 4 different measurement sets, with an average length of the maximal core-microbiome of $6.65 \pm 1.1$ taxa. The core-microbiome with the highest abundance (0.23) is $\{0,1,2,4\}$. In artificial measurements, the maximal abundance rank within the core-microbiome is between 14, occurring in 6 measurement sets, and 16, occurring in 65 measurement sets.

**Implication of bacterial interactions** As edges reflect upon rising taxa sets due to the properties of chemical organization theory certain reaction rules may be implied. Since it can not be ascertained that a subset is self maintaining there exist two possibilities for the reaction rules. One way is to say the taxa being present in the subsets of the considered set is needed to maintain the newly occurring taxa in this set. The other way is that the newly occurring taxa in any of the supersets are needed to maintain the newly occurring taxa in the considered set. Off course this can only be the case if the considered set itself is not self-maintaining. Lastly, it should be mentioned to be aware of the fact that there is a chance that every newly occurring taxon is potentially inflow.

Another way is to look at the correlation of taxa sets rather than individual taxa (see Supplement Fig. S2.2). This yields the possibility to respect the context of the taxa leading to more weak correlations in the upstream wells H41 and H43 and fewer but stronger correlations in the downstream wells H51 and H52. This change in set correlations might reflect upon an increase in the impact of co-occurring taxa along the aquifer, which is inline with^4^. Changing inflow might have a great impact on the observed upstream taxa sets leading to less co-occurrences.

#### Additional organizations

Apart from intersection sets introducing new taxa set operations also revealed 75 additional organizations, meaning that nearly 40% of all organizations are additional organizations (see “Methods” ). Those additional organizations, representing potential further measurements are complementing the lattice of organizations (see Supplement Fig. S5.6). On this way also the taxa set of one data point namely well H52 at time point 5 was predictable.

**Figure 10:**
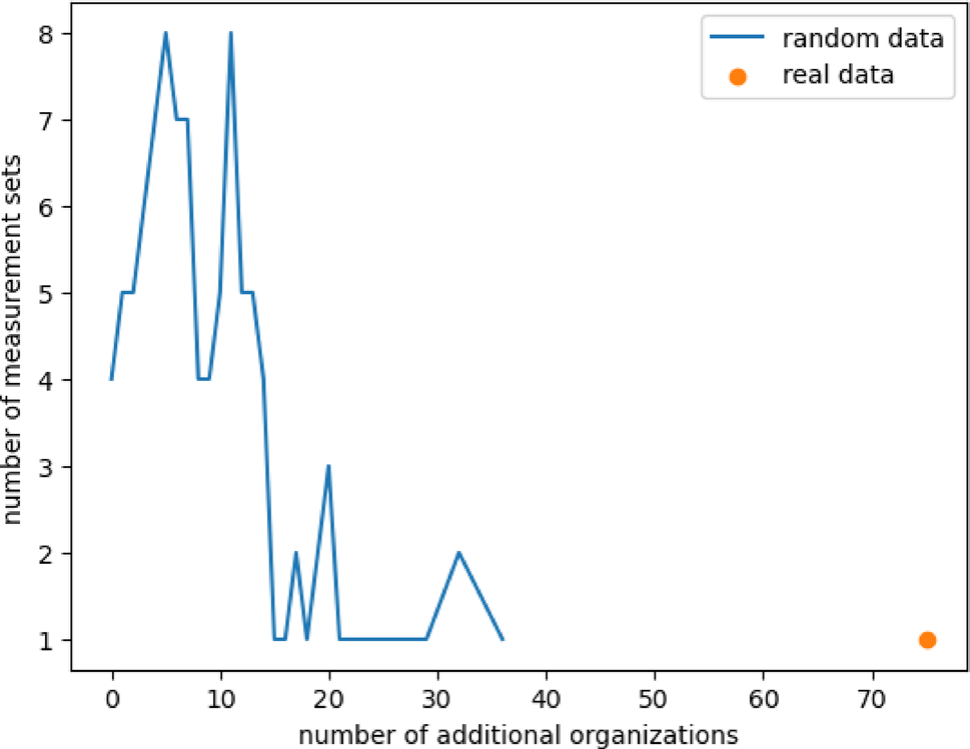
Additional organizations found in artificial measurement sets. Depicted is the number of measurement sets (vertical axis) that had a certain number of additional organizations (horizontal axis). The comparison between random data (blue) and real data (orange) shows that random measurement sets are less likely able to produce additional organizations with maximal 36 additional organizations compared to 75 additional organization in the real data.

**Differences to artificial measurements** In artificial measurement sets it was never possible to predict any measurement. This might be due to the relatively low number of $13.32 \pm 8.11$ computable organizations(see Fig. 10).

Even though there are 7 artificial measurement sets having at least 30 additional organizations. But even the highest amount of 43 additional organizations are not even close to the 75 additional organizations in the actual measurements. The number of different taxa in a single artificial organization is thereby ranging from 18 to 35 with the maximum being at 22 with 201 occurrences. In contrast, the additional organizations derived from the actual measurements range from 19 to 30 different taxa with the peak being 27 different taxa occurring 12 times.

**Detailed set connections on an individual specie set**

**Figure 11:**
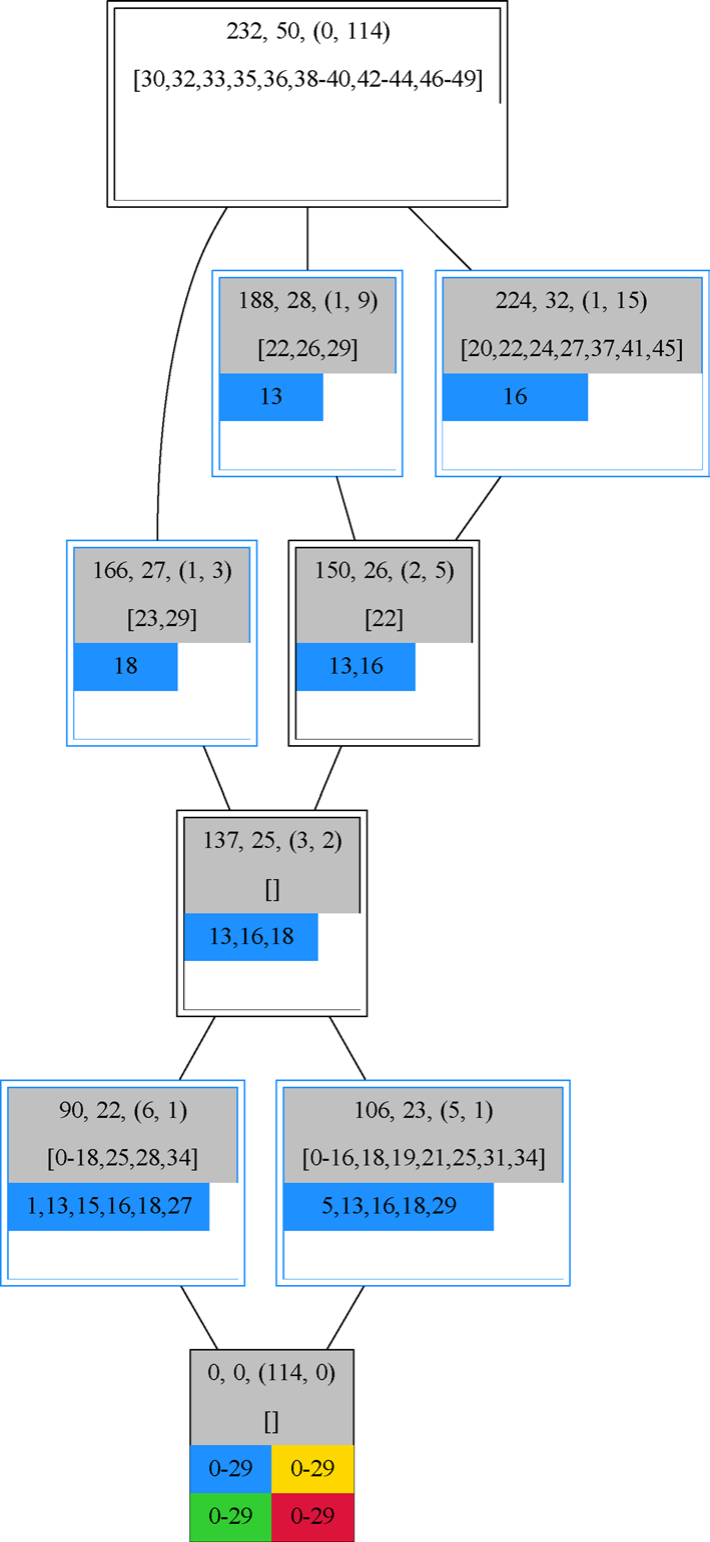
An additional organization in the lattice is recognizable on the gray background, indicating a self-maintaining set, and its black border, indicating the set was not a complete measurement. Set relations of the additional organization 137 (central node), which is most likely only observable in well H41 (blue). The difference to the two measurements below are in total 5 taxa with taxa 17 and 28 for set 106 and taxa 19, 21 and 31 for set 90 leads to a decrease from a total of 8 time points (5 and 6 respectively) down to 3 time points (13, 16 and 18).

Thanks to the color highlighting of the different wells it is easy to see in which wells such an additional organization is more likely to be observed. In the case of taxa set 137 this additional organization will most likely only be observable in well H41 (see Fig. 11). At the same time, the Hasse diagram reveals that measurements of well H41 have taxa 0-19, 21, 25, 28, 31, and 34 in common.

**Path of origins**

**Figure 12:**
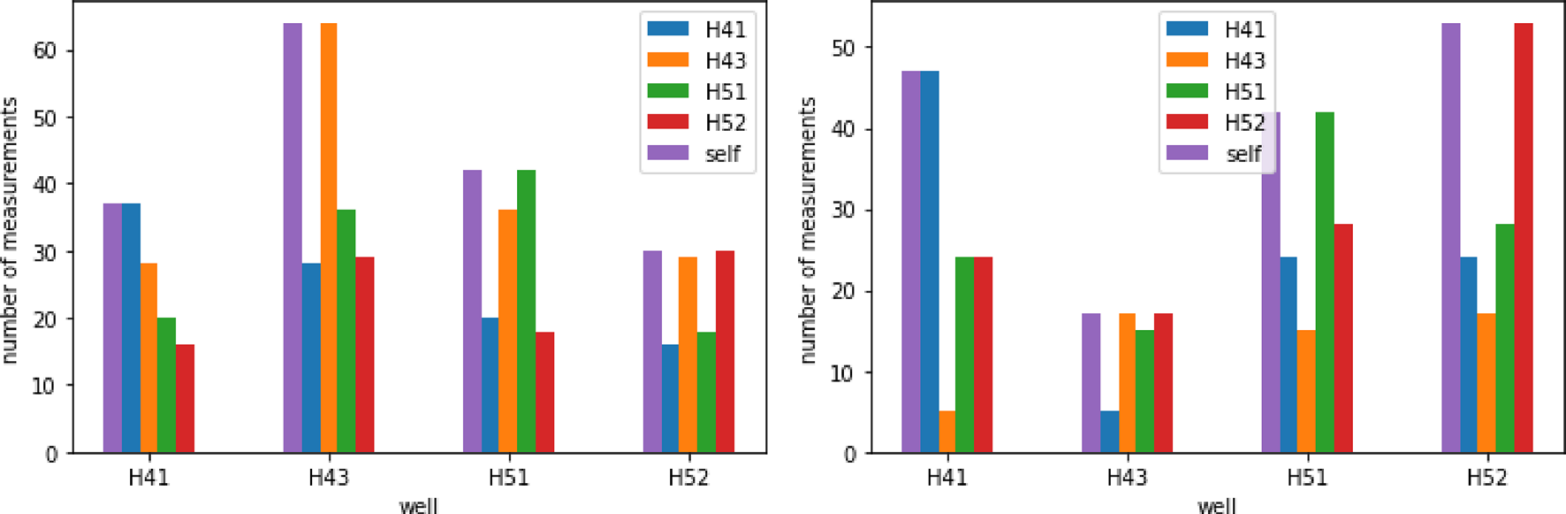
Number of additional organizations which are subsets (left) and supersets (right) of measurements belonging to the different wells. Purple bars show the total amount of organizations having a superset (left) or subset (right) measurement in the corresponding well on the x-axis. The blue, yellow, green, and red colored bars correspond to the portion of organizations which have at the same time a superset (left) or subset (right) measurement in the considered well respectively.

Identifying the highlighted fields of all additional organizations reveals that the large majority is a subset of taxa sets measured in the well H43 (see Fig. 12). As there are only 13 organizations which possess no superset in well H43 the number of additional organizations also possessing a superset in any other well is not that different compared to the total amount of that other well. The greatest difference occurs for well H41, with 10 organizations, indicating that the 13 missing additional organizations which are not included in any taxa set measured in well H43 are most likely included in a taxon set of well H41. On the other hand, it is observable that almost every additional organization which is a subset of a measurement in well H52 is also a subset of well H43 possibly hinting towards their connection. But well H41 and Well H51 also having a connection do not possess such a high number of shared additional organizations, which also may be due to their overall lower amount of additional organizations. If on the other hand, the additional organizations which are a superset of at least one measurement of a certain well were counted the most amount of organizations are found in well H52 (see Fig. 12). The highest amount of subset measurements is reached for well H52 whereas the lowest amount is observable for well H43. Like the superset measurements (Fig. 12) every organization having a subset in well H43 has also a subset in well H52. Well H41 and well H51 on the other hand share both ways only half of their additional organizations with each other.

**Figure 13:**
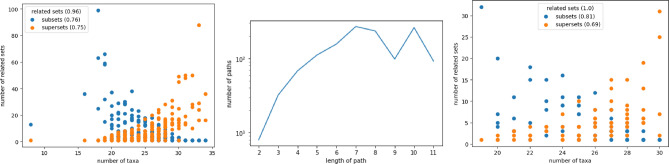
Connectivity of the lattice considering measurements along with additional organizations, left and middle, and solely additional organizations, right. Left and right have to be compared to Figure 7. The relatedness of the data rises under the consideration of additional organizations as they highlight similarities of different measurements that at the same time summarize the taxa composition of other measurements. The graph in the middle compared to Figure 8 underlines that enriched connectedness of the taxa sets by the enormous path elongation. There are even high amounts of paths possessing a follow-up of up to eleven measurements or additional organizations.

**Elevation of set connectivity and up-building specie sets ** In total those additional organizations lift the relatedness of the taxa sets by not only increasing the number of related sets of individual organizations but also by increasing the path lengths (see Fig. 13).

Now the most connected sets are related to over 80 other organizations. Compared to the measurements alone with 50 and 33 as the highest numbers of related sets it is nearly doubled(cf. Fig. 7). This great increase reflects the nature of those additional organizations as they elucidate the common sets but are unable to reveal taxa sets which possess combinations offside the measurements. Quit remarkable is the great gain in path lengths from 6 to 11 along with enormous changes in their amounts (cf. Figs. 8,13). Interestingly the drop formerly being at path length 4 seemed now be shifted towards a path length of 9. Longer paths in the lattice are a great opportunity as the consecutive sets following one path enlighten not only which taxa can be added to a certain set but also their ordering. As the additional organizations elongate the path in the middle the addable taxa sets shrink in size but grow in numbers. So that the resulting ordering is more fine-grained.

Concentrating now on the additional organizations which range from 19 to 30 different taxa allows for a more detailed view on the properties of those organizations. As they are constructed from the original measurements via set operations it is not surprising that they are all connected (see Fig. 13). Contrary to considerations respecting the measurements the highest connections are now indeed for the sets with the highest and lowest taxa diversity. Another interesting fact is that here opposed to the measurements is no gap between the highest number of related super and subsets (cf Fig. 7). Still, it remains that it is overall more likely to be a sub than a superset indicated by 81% of the additional organizations being a subset compared to 69% being a superset.

### Virtual simulated experiments

**Figure 14:**
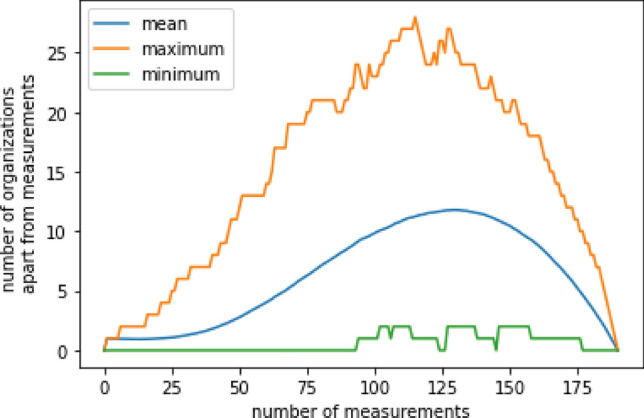
Number of possible additional organizations upon insertion of measurements (here: all inferred organizations, including the empty set) from 1000 repeats. At each step, a random organization from the list of all previously computed organizations is chosen. Through set operations between those selected organizations, we potentially know of other organizations before we observe them. Here curves of minimal (green), average (blue), and maximal (orange) numbers of additional organizations, highlight a region from 90 - 175 measurements possessing a high possibility of at least one with up to a maximum of 29 additional organizations, being not even half of the 75 original additional organizations. The maximum average at around 120 - 130 measurements is beyond our number of 114 samples.

Since measurements are not complete, the computation of additional organizations is an important opportunity to reveal additional sustainable bacterial communities. The number of additional organizations is thereby dependent on the number of measurements as a representation of the portion of the total amount of organizations, here actual measurements, 114, and original additional organizations, 75. (see Fig. 14).

Random draw without replacement suggests rather low numbers of additional organizations similar to random measurements. As additional organizations have to be at the same time cut and union of at least two different measurements they are dependent on the measurement selection. The average maximum being at around 125 to 130 (see Fig. 14, blue line) indicates that 66 to 69 % of the organizations have to be known to get the most amount of additional organizations. Still, it is observable that even with 175 measured organizations and 5 additional organizations not the total amount of 189 organizations can be retrieved. Even though additional organizations can extend the lattice of measurements the complete lattice of organizations remains unknown.

## Conclusion

While classical methods like PCoA project whole measurements to points in a 2D plane where the actual composition of the points remains hidden, our method, in contrast, is able to reveal those taxa compositions. Even though only the presence and absence of a taxon is considered, a hierarchical structure of the data is revealed. Opposing to the Bray-Curtis-Distances in PCoA, the differences between the two measurements in terms of taxa composition can be seen directly and therefore treated individually for each taxon.

With the added information contained in the nodes (see Fig.  3) of the lattice it is possible to distinguish taxa combinations preferring a certain well and sometimes even exhibiting temporal patterns from others that are distributed more equally over wells and time points.

The new method connecting COT and FCA reduces significantly the amount of intersection sets, here from 15390 to only 117 interesting intersection sets; of those 75 organizations (potentially persistent sets) and 42 sets with new taxa, denoting the smallest context a taxon is appearing in. This allows to notice and describe all reoccurring combinations of taxa.

These combinations are arranged in a Hasse diagram, which visualizes the linkages between all subsets of taxa including the measurements and the intersection sets. The similarities and also dissimilarities across space and time are highlighted and integrated into the hierarchical structure of the community.

For each individual set (e.g., Fig. 9) the different possibilities, to which this set of taxa can be extended by the union of measurements or reduced by the intersection of measurements, can be extracted. If the complete Hasse diagram is too large as in our study it is possible to zoom in by selecting an individual set, e.g., Fig. 9. This individual view displays all measurements, where the selected set appears (supersets) as well as all parts of that measurement (subsets) that appear in other measurements. This in turn can support reconstructing a bacterial interaction network model in the future. As the hierarchical structure is based on COT, it is only able to reflect upon the positive and neutral interactions of the network. But this only affects the network reconstruction and leaves the here presented hierarchical structure unaffected. Because if two taxa are measured together they possess, according to our assumptions, a possibility to coexist in a community.

The core-microbiome, formerly a single set of taxa, where only each individual taxa has to be present in at least 90 % of the measurements, is now dividable into different core-microbiomes respecting the context in which those taxa occur. The different core-microbiomes consisting of an individual set of core taxa yield the possibility to connect the appearance of certain sets of core taxa to certain environmental conditions, here space, and time. The next step would be to look at the causation of shifts from one core-microbiome to the next.

Taxa sets that are intersections as well as unions of measurements have the properties of organizations and are thus predicted as potentially persistent bacterial communities, which can be tested in subsequent experiments. The inclusion of environmental conditions (here, space and time) might help to better understand when to expect such an additional persistent community.

The randomization of the data used in this work caused a loss of structure and a reduction of the number of additional organizations indicating that the structures of the microbial community revealed by the new method are significant.

The technique presented in this work can be transferred to any other model consisting of self-replicating agents and is not restricted to bacterial communities. In summary it is now possible to highlight the set connections of the individual measurements such that the formation of a taxa combination is now directly linked to certain environmental conditions as well as there are now directly visible different core microbiomes. This leads to a hierarchical structure revealing the possible dependencies of taxa combinations on environmental conditions and taxa interactions.

### Supplementary Information

Supplementary Information.

## Data Availability

The data was generated as explained here https://doi.org/10.1016/j.watres.2021.117290 and can be reached via https://git.uni-jena.de/ne78xoy/focusedfca.
