# Supplementary material for: Revealing the hierarchical structure of microbial communities
Source: Sci Rep. 2024 May 16;14:11202. doi: 10.1038/s41598-024-61836-3 (PMC11099180; doi:10.1038/s41598-024-61836-3)
Supplement: Supplementary file 1 — Supplementary Information. [file 41598_2024_61836_MOESM1_ESM.pdf]

Supplemantary Information

S1 Artificial measurements

100 sets of 120 artificial measurements based on the relative taxa abundance of each well were generated. So that each set has 30 measurements of each well. Through chi-square tests between artificial and actual measurements of the individual wells, we see that the artificial measurements based on well-specific taxa abundance have a minimal p-value of 0.79 for well H43. There is no significant change in the well-specific taxa abundance between actual and artificial measurements. Looking at the average number of different taxa occurring in a single measurement there is no significant change between artificial and actual measurements for the individual wells as the minimal p-value is $p = 0.851$ reached for well H52. The diversity

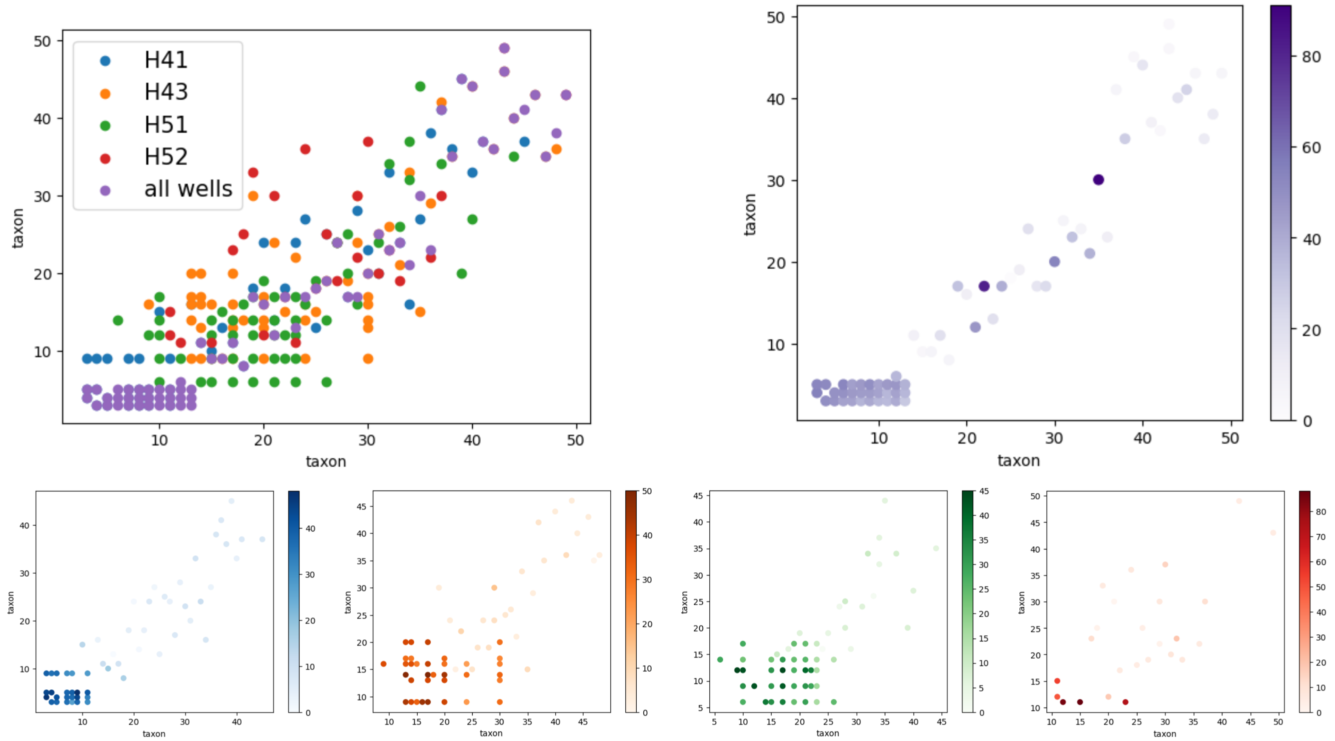

Figure S1.1. Maximal co-occurrence for all taxa. Top Left: Y-axis depicts the taxon which has the maximal correlation coefficient with the taxon on the X-axis. Rest: Number of replicated taxa with maximal co-occurrence in 100 artificial measurement sets. Depicted are the individual wells H41 in blue, H43 in orange, H51 in green and H52 in red (bottom) and all wells taken together in purple (top right). The color intensity represents the reproducibility of maximal co-occurrence in the artificial measurements.

of the artificial measurements is thereby ranging from 16 to 35 different taxa. A two sample Kolmogorov-Smirnov test for the frequency distribution between the artificial and actual measurements of each individual well, with a minimal p-value of 0.716 for well H51, reveals reproducibility through abundance-based taxa selection. Also, the Spearman rank-order correlation coefficient for the artificial measurements of the individual wells reveals a high correlation to the rank order of the taxa in the actual measurements with the lowest correlation being 0.85 with a p-value of $4.84e-15$ in well H52.

But the maximal co-occurring taxa are not often reproducible by generating artificial measurements based on relative taxa abundances (see Fig. S1.1). Excluding due to their high abundance taxa 0 - 10 reveals that there are in total 11 maximal co-occurring taxa which are also detectable by artificial measurements in at least 20% of the artificial measurements. Even though the maximal co-occurrence for individual wells can not be reproduced via our artificial measurements some overall co-occurrences are very likely reproduced. It is observable that overall taxon 35 possesses a maximal co-occurrence with taxon 30 in 95 of the 100 artificial measurement sets. Also, taxon 22 with taxon 17 was reproduced 75 times. So they can only be measured together because their individual taxa abundances force them together. Those exceptions as they likely occur also randomly have to be excluded from possible positive interactions.

S2 Set correlations

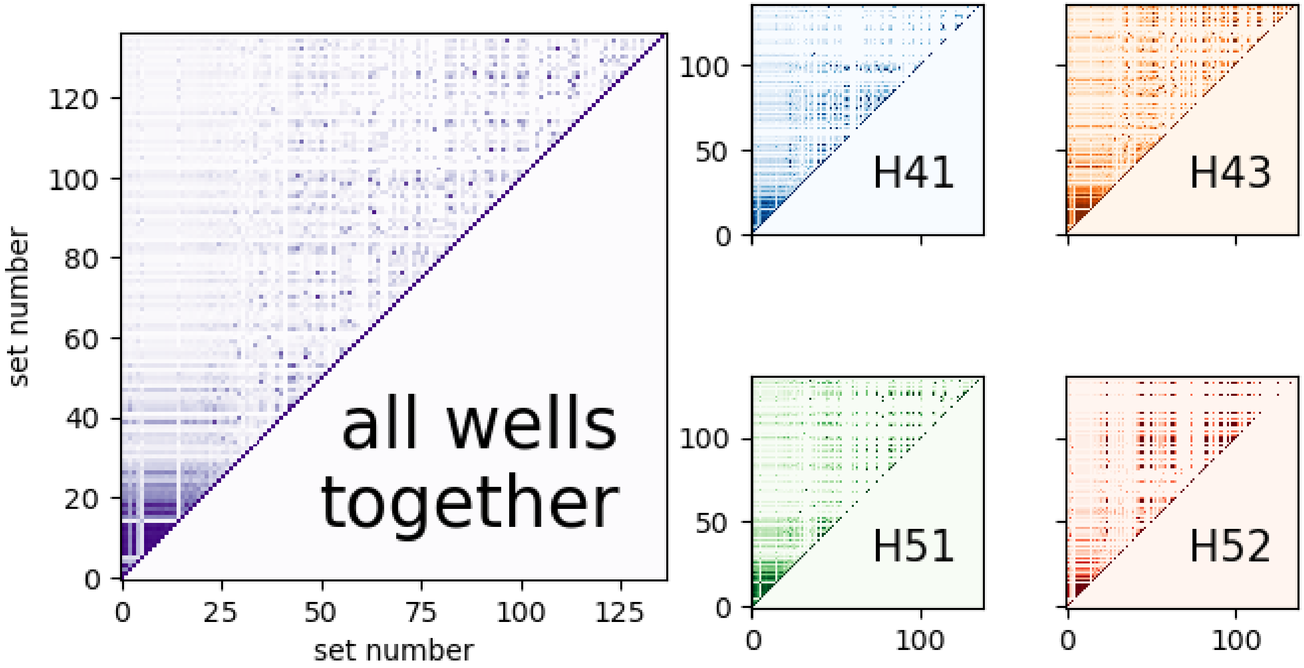

Figure S2.2. Co-occurrence of taxa sets having a maximal of 25 different taxa, to include the maximal intersection set. The set number corresponds to the set number of the Hasse diagram.

S3 All measurements

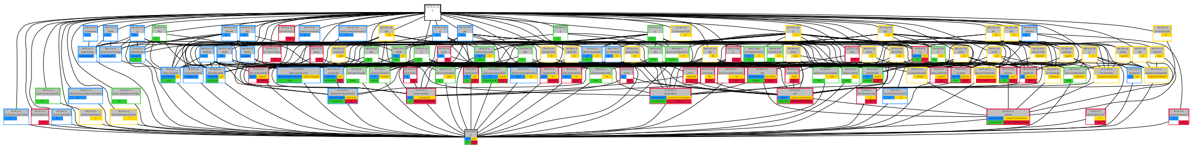

Figure S3.3. Ordering of all measurements as largest subsets. A node representing a distinct set of taxa consists of an identification number, followed by taxa count and the number of sets it is present in, in parenthesis, and a list of taxa that were not present in any of its subsets. The bottom of each node consists of 4 fields corresponding to the four different wells H41(green), H43(blue), H51(red), H52(yellow) containing the measurements it is part of.

S4 All measurements with subsets introducing new taxa

S5 All organizations

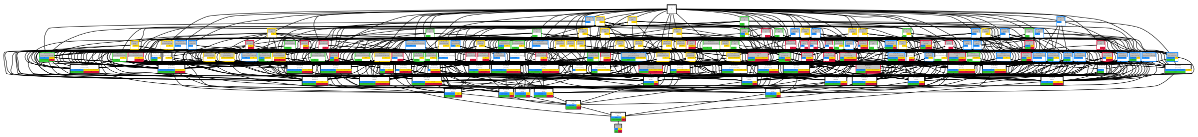

Figure S4.4. Ordering of all closed sets introducing new taxa as largest subsets.

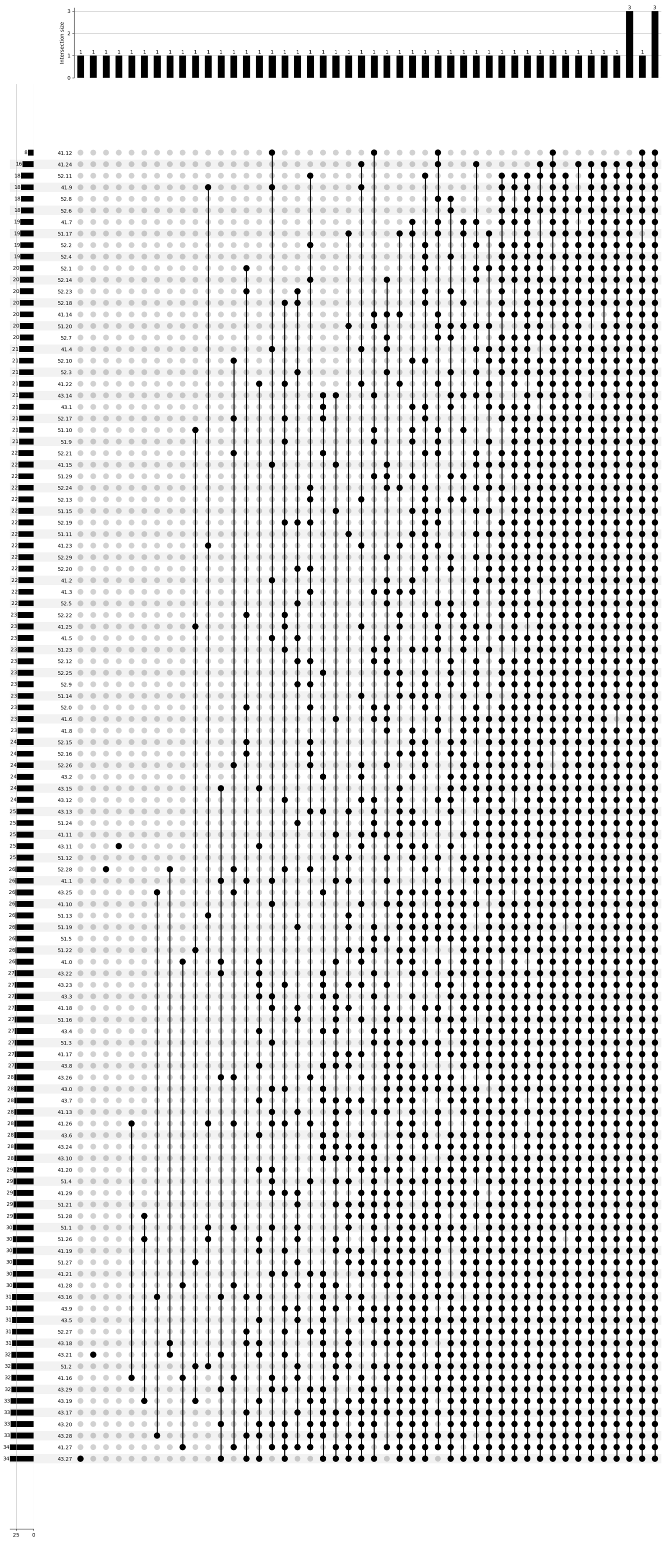

Figure S4.5. Upsetplot of all measurements: Combination of different taxa sets (x-axis) leading to different measurements (y-axis).

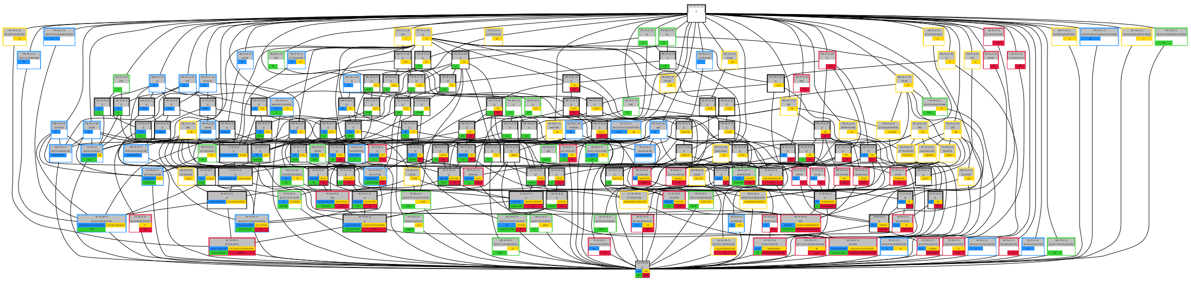

Figure S5.6. Ordering of all identified organizations as largest subsets.
